# Clinical application of BALF-mNGS in immunocompromised and immunocompetent patients with suspected invasive pulmonary aspergillosis: differentiating infection from colonization, microbiome features, and clinical impact

**DOI:** 10.1128/spectrum.01045-26

**Published:** 2026-08-28

**Authors:** Junjie Zhao, Xinxin Chen, Xinyi Wang, Yidan Cui, Jiancheng Zhuge, Honglong Fang, Zhidan Hua

**Affiliations:** 1 Emergency and Critical Care Center, Department of Emergency Medicine, Zhejiang Provincial People's Hospital (Affiliated People's Hospital), Hangzhou Medical College 74678, Hangzhou, Zhejiang, China; 2 Hangzhou Normal University 26494, Hangzhou, Zhejiang, China; 3 Department of Critical Care Medicine, Quzhou TCM Hospital at the Junction of Four Provinces Affiliated to Zhejiang Chinese Medical University, Quzhou, Zhejiang, China; 4 Department of Critical Care Medicine, The Quzhou Affiliated Hospital of Wenzhou Medical University, Quzhou People’s Hospital 91619, Quzhou, Zhejiang, China; 5 Plmonary and Critical Care Medicine, The Quzhou Affiliated Hospital of Wenzhou Medical University, Quzhou People’s Hospital 91619, Quzhou, Zhejiang, China; Universidade de Sao Paulo Campus de Ribeirao Preto, Sao Paulo, Brazil

**Keywords:** *Aspergillus*, metagenomic next-generation sequencing, immunocompromised patient, lung microbiota, bronchoalveolar lavage fluid

## Abstract

Invasive pulmonary aspergillosis (IPA) not only causes high morbidity and mortality, especially in immunocompromised patients, but also occurs in immunocompetent individuals. Differentiating infection from colonization is challenging, and bronchoalveolar lavage fluid metagenomic next-generation sequencing (BALF-mNGS) may aid diagnosis, microbiome profiling, and clinical assessment. We retrospectively analyzed patients with suspected IPA who underwent BALF-mNGS between December 2021 and March 2025. Patients were classified by immune status. IPA diagnosis was based on EORTC/MSGERC 2020 criteria in immunocompromised patients, while immunocompetent cases were adjudicated using an integrated clinical, radiological, and microbiological assessment. A total of 178 patients were finally included and classified according to immune status into an immunocompromised group (*n* = 77) and an immunocompetent group (*n* = 101). *Aspergillus*_ reads per ten million (RPTM) values were significantly higher in infection versus colonization cases in both groups, with optimal cut-offs of 24 (gray zone: 22–60) for the immunocompromised group and 33 (gray zone: 17–48) for the immunocompetent group. Microbiome analysis revealed distinct community structures between infection and colonization groups, with *Aspergillus* remaining significantly enriched after false discovery rate (FDR) correction in immunocompromised patients, while no taxa remained significant after FDR correction in immunocompetent patients. BALF-mNGS guided antifungal therapy, avoiding unnecessary treatment in colonized patients, and higher Aspergillus_RPTM was associated with increased 90-day mortality in immunocompromised patients. BALF-mNGS may accurately distinguish *Aspergillus* infection from colonization and reveal microbial shifts, particularly in immunocompromised patients. It can guide targeted antifungal therapy, avoid unnecessary treatment, and higher Aspergillus_RPTM was associated with 90-day mortality in immunocompromised patients, suggesting its potential value for risk stratification.

**IMPORTANCE:**

BALF-mNGS with quantitative thresholds improves differentiation between *Aspergillus* infection and colonization. It reveals distinct lung microbiome patterns under different immune statuses, extending beyond simple pathogen detection. Higher *Aspergillus* burden is associated with worse outcomes in immunocompromised patients, suggesting its potential value for risk stratification.

## INTRODUCTION

Invasive pulmonary aspergillosis (IPA) has traditionally been regarded as a disease of severely immunocompromised patients, whereas the detection of *Aspergillus* in immunocompetent individuals has generally been considered colonization rather than true infection (1, 2). More recently, however, the incidence of IPA has been rising in both immunocompetent and immunocompromised individuals (3). This trend is likely multifactorial, driven by advances in therapies for malignancies and rheumatologic disorders, thereby increasing indications for solid organ and hematopoietic transplantation, HIV infection, prolonged intensive care unit (ICU) stays, and the impact of climate change (4, 5), often leading to delayed recognition and high mortality (6). The nonspecific clinical presentation of IPA further complicates diagnosis, particularly in rapidly progressing non-neutropenic cases with poor prognosis, underscoring the importance of early and accurate detection (7, 8).

Although histopathological examination and culture are regarded as the gold standard for diagnosing IPA, histopathology is invasive, requires adequate tissue sampling, and is often difficult to obtain in critically ill patients (9). Conventional diagnostic methods for IPA mainly include fungal culture, biomarker assays, such as (1,3)-β-D-glucan and galactomannan (GM), polymerase chain reaction (PCR)-based techniques, and chest computed tomography (CT) imaging. However, all have limitations: fungal culture is slow and insensitive (1); biomarker assays are affected by antifungal or antibiotic therapy and delayed peak levels (10); PCR, though sensitive and specific, is not routinely available in clinical practice in China for the diagnosis of *Aspergillus* infection (11); and CT findings are nonspecific, limiting diagnostic accuracy (12).

In light of these limitations, with the advent of high-throughput sequencing technologies, metagenomic next-generation sequencing (mNGS) has emerged as a promising tool for the diagnosis of IPA (13–15). However, most existing studies have primarily focused on the diagnostic performance of mNGS for IPA and its role in guiding antibiotic regimen adjustments, while relatively few have explored its potential in distinguishing *Aspergillus* colonization from true infection. To date, only one study by Jiang et al. (16) has systematically evaluated the clinical performance of mNGS in this context. In their analysis of bronchoalveolar lavage fluid (BALF) samples from immunocompromised patients, they demonstrated that an *Aspergillus* read count threshold of 23 achieved an area under the curve (AUC) of 0.894 for differentiating infection from colonization, underscoring both the promise of mNGS and the rarity of research in this area. Notably, their study was limited to immunocompromised patients, leaving it unclear whether these findings apply to immunocompetent individuals.

Building on this gap, the present study aimed to further assess the performance of quantitative BALF-mNGS in distinguishing *Aspergillus* infection from colonization among patients with Aspergillus-positive BALF-mNGS results, characterize pulmonary microbiome features, and explore the prognostic value of mNGS findings among patients with Aspergillus-positive BALF-mNGS results stratified by immune status, which represents a novel aspect of our work.

## MATERIALS AND METHODS

### Patients

This multicenter retrospective study included patients with suspected IPA detected by BALF-mNGS results who were admitted to hospitals between December 2021 and March 2025. The study protocol was approved by the Ethics Review Committee of Quzhou People’s Hospital (No. 2025-117), and the patients were anonymized for analysis.

**Fig 1:**
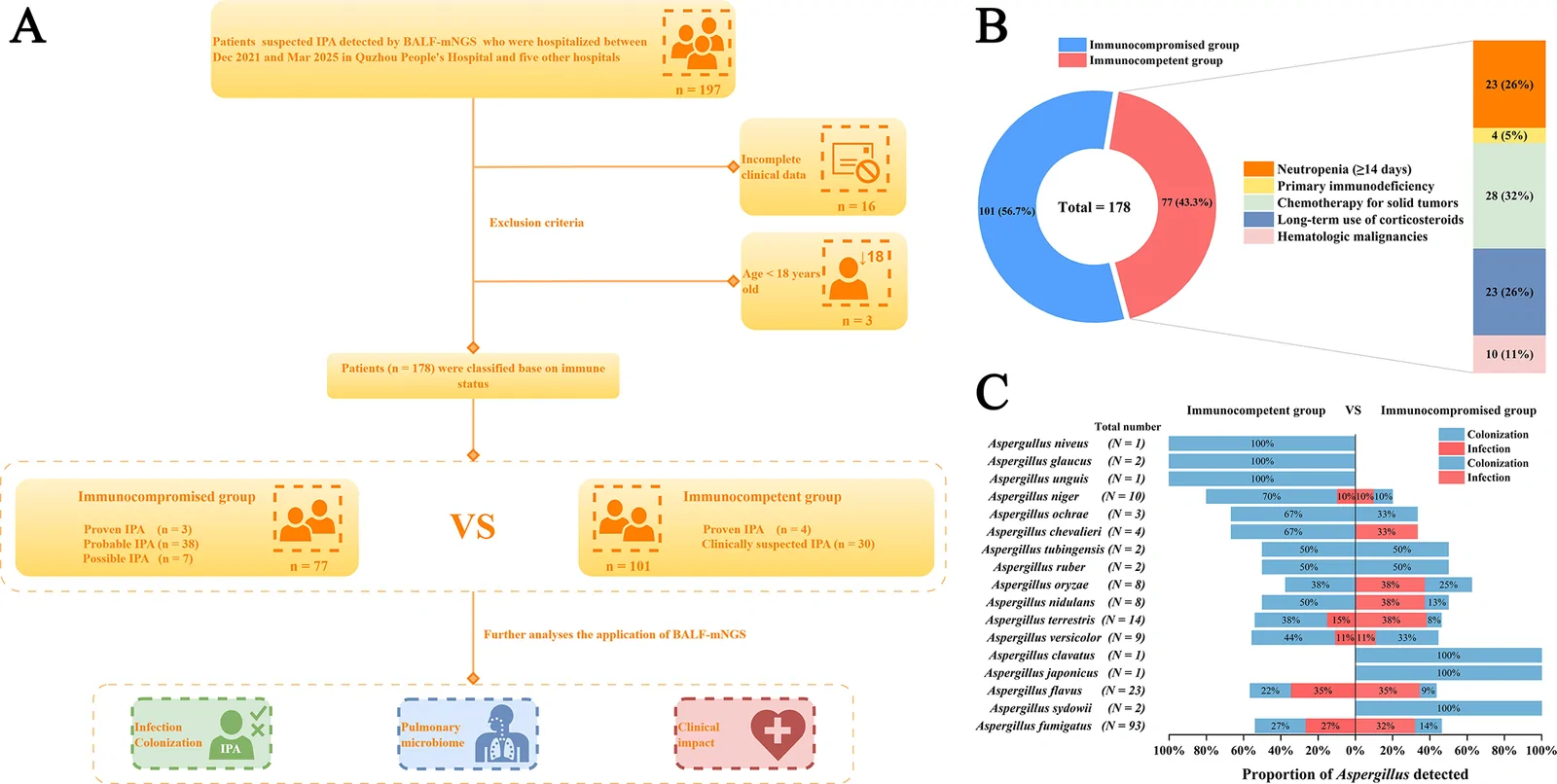
(**A**) Study flowchart; (**B**) distribution of immune status and immunocompromised subgroups; and (**C**) distribution of *Aspergillus* colonization and infection between the two groups.

A total of 178 patients were identified for analysis, and their clinical data were collected (Fig. 1A). The inclusion criteria were as follows: (i) patients suspected of having IPA underwent BALF-mNGS testing, and *Aspergillus* was detected by mNGS; and (ii) age ≥ 18 years old. The exclusion criteria included (i) age < 18 years old; and (2) incomplete clinical data. Patients were classified into the immunocompromised and immunocompetent groups according to their immune status. Patients were considered immunocompromised if they met one or more of the following criteria (17, 18): (i) hematologic malignancy; (ii) confirmed primary immunodeficiency disorders; (iii) neutropenia or administration of chemotherapy for solid tumors within the preceding 3 months; (iv) prolonged systemic corticosteroid use specified as a prednisone-equivalent dose ≥0.3 mg/kg/day for a duration of ≥3 weeks; (v) current or recent treatment with antirheumatic drugs, biologic immunomodulators, or other immunosuppressive agents; and (vi) history of solid organ transplantation.

### Criteria for the diagnosis of IPA

In immunocompromised patients, IPA diagnosis was established according to the European Organization for Research and Treatment of Cancer and the Mycoses Study Group Education and Research Consortium (EORTC/MSGERC) consensus definitions (10):

1. Proven IPA was defined by histopathological, cytological, or direct microscopic evidence of hyphae in specimens obtained through biopsy or needle aspiration, together with demonstration of tissue damage.
2. Probable IPA required the simultaneous presence of at least one host factor, compatible clinical manifestations, and mycological evidence. Mycological evidence was considered positive when any of the following conditions were met: (i) culture or histopathological examination positive for *Aspergillus*; (ii) galactomannan (GM) assay interpreted as positive with thresholds of serum/plasma ≥1.0 or BALF ≥1.0 or alternatively serum/plasma ≥0.7 combined with BALF ≥0.8; (iii) two or more positive polymerase chain reaction (PCR) results from plasma, serum, whole blood, or BALF, or at least one positive result from both blood and BALF; or (iv) isolation of *Aspergillus* from respiratory specimens, including sputum, BALF, bronchial brush, or aspirates.
3. Possible IPA was considered when host factors and clinical or radiological findings were consistent with IPA but no mycological evidence was documented.

Proven IPA was diagnosed based on EORTC/MSGERC (histopathological evidence) and applicable to both immunocompromised and immunocompetent patients. For immunocompetent patients without classical host factors, IPA diagnosis was based on an integrated assessment of clinical manifestations, radiological findings, conventional microbiological evidence, and expert adjudication.

Cases were classified as colonization when *Aspergillus* was detected in respiratory specimens, but the patients did not meet the diagnostic criteria for IPA or other clinically significant Aspergillus-related airway diseases, such as allergic bronchopulmonary aspergillosis (ABPA) or *Aspergillus* bronchitis, after comprehensive assessment of host factors, clinical manifestations, radiological findings, and conventional microbiological evidence. The adjudicators were blinded to BALF-mNGS results, including Aspergillus_RPTM values, which were not incorporated into the reference standard or used for final case classification. In cases of disagreement, a senior physician reviewed all available clinical information and made the final decision.

### Sample collection and conventional microbiological tests

Fibreoptic bronchoscopy was used to obtain BALF samples for both mNGS and conventional microbiological tests (CMTs). CMTs included BALF culture, GM test, (1,3)-β-D-glucan test, and PCR for *Aspergillus* detection. Histological biopsy of lung lesions was performed in a subset of patients via bronchoscopy or chest computed tomography (CT)-guided percutaneous lung biopsy. Detailed results of available conventional microbiological tests are summarized in Table S1.

### Metagenomics next-generation sequencing and bioinformatics analyses

BALF sample (1.5–3 mL) was obtained via bronchoscopy and centrifuged at 12,075 × *g* for 5 min at 4°C. A 500 μL aliquot of the supernatant was used for nucleic acid extraction. DNA was isolated using a universal pathogen enrichment kit (PathoXtract, WillingMed, Beijing, China), and RNA was purified with a virus DNA/RNA extraction kit from the same manufacturer following the recommended protocols. For library preparation, DNA-only analyses were performed using the Illumina DNA Prep (M) Tagmentation Kit, whereas RNA samples were first reverse-transcribed into complementary DNA (cDNA) with the SuperScript Double-Stranded cDNA Synthesis Kit (Invitrogen), and then pooled with the DNA material. Library quality and concentration were assessed with a Qubit fluorometer and an Agilent 2100 bioanalyzer. Sequencing was carried out on the Illumina NextSeq 550Dx platform, with each sample generating at least 20 million reads.

Raw sequencing data were processed through a standardized bioinformatics pipeline. Reads of low quality, adaptor sequences, highly repetitive fragments, and short inserts were filtered out using Trimmomatic v0.40 (19). High-quality reads were aligned to the human reference genome GRCh37 (hg19) using Bowtie2 v2.4.3 (20) to eliminate host-derived sequences. The remaining non-human reads were compared with a curated microbial reference database containing more than 24,000 pathogens. Taxonomic classification was performed with Kraken2 v2.1.0 (21), and pathogen identification was determined based on species-specific reads normalized as RPTM (reads per ten million, defined as the number of pathogen-specific reads per ten million total reads). All mNGS analyses were conducted in the same laboratory (WillingMed, Beijing, China).

### Clinical impact of BALF-mNGS

The clinical impact of BALF-mNGS was assessed by evaluating its influence on antimicrobial regimen adjustments and patient outcomes. In our center, BALF-mNGS results were interpreted conservatively and were not used in isolation to guide therapy. Antimicrobial regimen adjustments were considered after integrating BALF-mNGS findings with conventional microbiological test results and the overall clinical context.

Patients were classified into four categories based on antimicrobial regimen adjustments:

1. Antifungal initiation: patients without empirical antifungal therapy, in whom the integrated results confirmed invasive pulmonary aspergillosis (IPA), leading to initiation of antifungal treatment.
2. Confirmed: patients who had received empirical antifungal therapy, and the integrated results supported the diagnosis of IPA, leading to continuation of therapy.
3. Antifungal therapy not initiated: patients without empirical antifungal therapy, and the integrated results excluded IPA, so antifungal treatment was not initiated.
4. Excluded: patients who had received empirical antifungal therapy, but the integrated results excluded IPA, resulting in discontinuation of antifungal agents.

Furthermore, to better evaluate the prognostic implications of microbial load, we conducted a logistic regression analysis of Aspergillus_ (reads per ten million) RPTM for predicting 90-day mortality across different patient subgroups.

### Statistical analysis

Data were statistically analyzed using R software (version 4.4.1; R Foundation for Statistical Computing, Vienna, Austria). Figures were created using OriginPro (version 2025b; OriginLab Corp., Northampton, MA, USA). Continuous variables were tested for normality using the Kolmogorov–Smirnov test and expressed as mean ± standard deviation (SD), with categorical variables as *n* (%). For comparisons between two independent groups, an independent samples *t*-test was used for normally distributed data, and the Wilcoxon rank sum test was used for non-normally distributed data. For comparisons across multiple related samples, the Friedman test was applied. Categorical variables were compared using the *χ* test. Receiver operating characteristic (ROC) curve analysis was first performed to evaluate the predictive performance of RPTM values for IPA. The optimal cutoff value was determined using the maximum Youden index. Gray-zone analysis based on 1,000 bootstrap resamples was subsequently performed to identify the interval of diagnostic uncertainty surrounding the optimal cutoff (22). The gray zone corresponds to the range of RPTM values where either sensitivity or specificity did not reach 90%, indicating that infection and colonization could not be reliably distinguished using RPTM alone. Univariable logistic regression was performed to evaluate the association between log-transformed Aspergillus_RPTM and 90-day mortality across different patient subgroups. The α diversity measures were compared for significance using the Wilcoxon rank-sum test. The β diversity was calculated using the Bray–Curtis dissimilarity method, and principal coordinates analysis (PCoA) was used to visualize differences in microbial community structure. Analysis of similarities (ANOSIM) was performed to assess the statistical significance of group separation based on β diversity. Linear discriminant analysis effect size (LEfSe) was applied to identify taxa contributing to differences between groups. Taxa were considered discriminative when they met the predefined criteria of an LDA score > 2.0 and a LEfSe-derived *P* value < 0.05. To account for multiple comparisons, false discovery rate (FDR) correction was subsequently performed, and taxa with an FDR-adjusted *P* value < 0.05 were considered statistically significant.

## RESULTS

### Demographic and clinical characteristics of the patients

Between December 2021 and March 2025, a total of 197 patients suspected of having IPA were enrolled. After excluding 16 patients with incomplete clinical data and three patients under 18 years of age, 178 patients were ultimately included in the study (Fig. 1A). Patients were classified according to their immune status into an immunocompromised group (*n* = 77) and an immunocompetent group (*n* = 101). Based on the diagnostic criteria for IPA, the immunocompromised group included proven IPA (*n* = 3), probable IPA (*n* = 38), and possible IPA (*n* = 7). In immunocompetent patients, IPA cases were classified according to expert adjudication based on integrated clinical, radiological, and microbiological evidence, including proven IPA (*n* = 4) and clinically suspected IPA categories corresponding to varying levels of diagnostic certainty (*n* = 30) (Fig. 1A).

The immunocompromised group comprised 43.3% of all patients. The most common types of immunosuppression were chemotherapy for solid tumors (32%), long-term corticosteroid therapy (26%), and neutropenia (26%) (Fig. 1B). The most frequently detected *Aspergillus* species (spp.) in both groups were *Aspergillus fumigatus*, *Aspergillus flavus*, and *Aspergillus terrestris* (Fig. 1C).

As shown in Table 1, patients in the immunocompromised group were older compared with those in the immunocompetent group (70 [64–78] vs. 67 [56–73], *P* = 0.006). The prevalence of COPD was significantly higher in the immunocompromised group (22.08% vs. 9.90%, *P* = 0.025). Laboratory tests revealed that immunocompromised patients had lower platelet counts (153 [91–226] vs. 206 [138–259], *P* = 0.002), higher blood urea nitrogen levels (6.08 [3.94–10.20] vs. 5.09 [4.10–6.59], *P* = 0.043), and elevated procalcitonin levels (0.57 [0.10–10.25] vs. 0.10 [0.02–1.43], *P* = 0.001). Furthermore, a greater proportion of immunocompromised patients required ICU admission (41.56% vs. 26.73%, *P* = 0.038), and the 90-day mortality was significantly higher in this group (49.35% vs. 28.71%, *P* = 0.007). In addition, there was a trend toward a longer length of hospital stay in the immunocompromised group (10 [7–14] vs. 8 [6–15], *P* = 0.056).

**TABLE 1:** Demographic and clinical characteristics of the patients^*a*^^,^^*b*^

	Immunocompromised group (*N* = 77)	Immunocompetent group (*N* = 101)	*P* value
Characteristics			
Age, years	70 (64–78)	67 (56–73)	**0.006**
Male, *n* (%)	49 (63.63%)	48 (47.52%)	0.332
Comorbidity, *n* (%)			
Hypertension	18 (23.38%)	23 (20.79%)	0.680
Diabetes	7 (9.09%)	7 (6.93%)	0.597
COPD	17 (22.08%)	10 (9.90%)	**0.025**
Laboratory parameters			
WBC, ×10^9^/L	7.62 (5.25–11.95)	7.90 (5.45–10.85)	0.687
Neutrophil, ×10^9^/L	5.00 (2.35–9.13)	5.62 (3.84–9.43)	0.196
Lymphocyte, ×10^9^/L	0.90 (0.50–2.20)	0.91 (0.47–1.75)	0.576
Hemoglobin, g/L	110 (98–125)	115 (101–127)	0.160
Platelet, ×10^9^/L	153 (91–226)	206 (138–259)	**0.002**
ALT, U/L	25.10 (14.80–50.40)	19.95 (13.68–34.90)	0.121
TBiL, μmol/L	11.20 (7.80–19.10)	10.60 (7.65–15.20)	0.276
BUN, mmol/L	6.08 (3.94–10.20)	5.09 (4.10–6.59)	**0.043**
Creatinine, μmol/L	67.10 (56.40–95.30)	61.80 (53.00–77.95)	0.097
Procalcitonin, ng/ml	0.57 (0.10–10.25)	0.10 (0.02–1.43)	**0.001**
CRP, mg/L	54.88 (10.02–160.72)	34.74 (4.59–110.54)	0.097
ICU admission, *n* (%)	32 (41.56%)	27 (26.73%)	**0.038**
LOHS, days	10 (7–14)	8 (6–15)	**0.056**
90-Day mortality, *n* (%)	38 (49.35%)	29 (28.71%)	**0.007**

^*a*^ Note: continuous variables are expressed as mean ± SD, and categorical variables as *n* (%). Abbreviation: WBC, white blood count; ALT, alanine aminotransferase; BUN, blood urea nitrogen; COPD, chronic obstructive pulmonary disease; CKD, chronic kidney disease; TBil, total bilirubin; CRP, C-reactive protein; and LOHS, length of hospital stays.

^*b*^ Bold text in Table 1 is used to highlight statistically significant P values (P < 0.05) for ease of reading.

### Diagnostic performance of RPTM values for IPA

**Fig 2:**
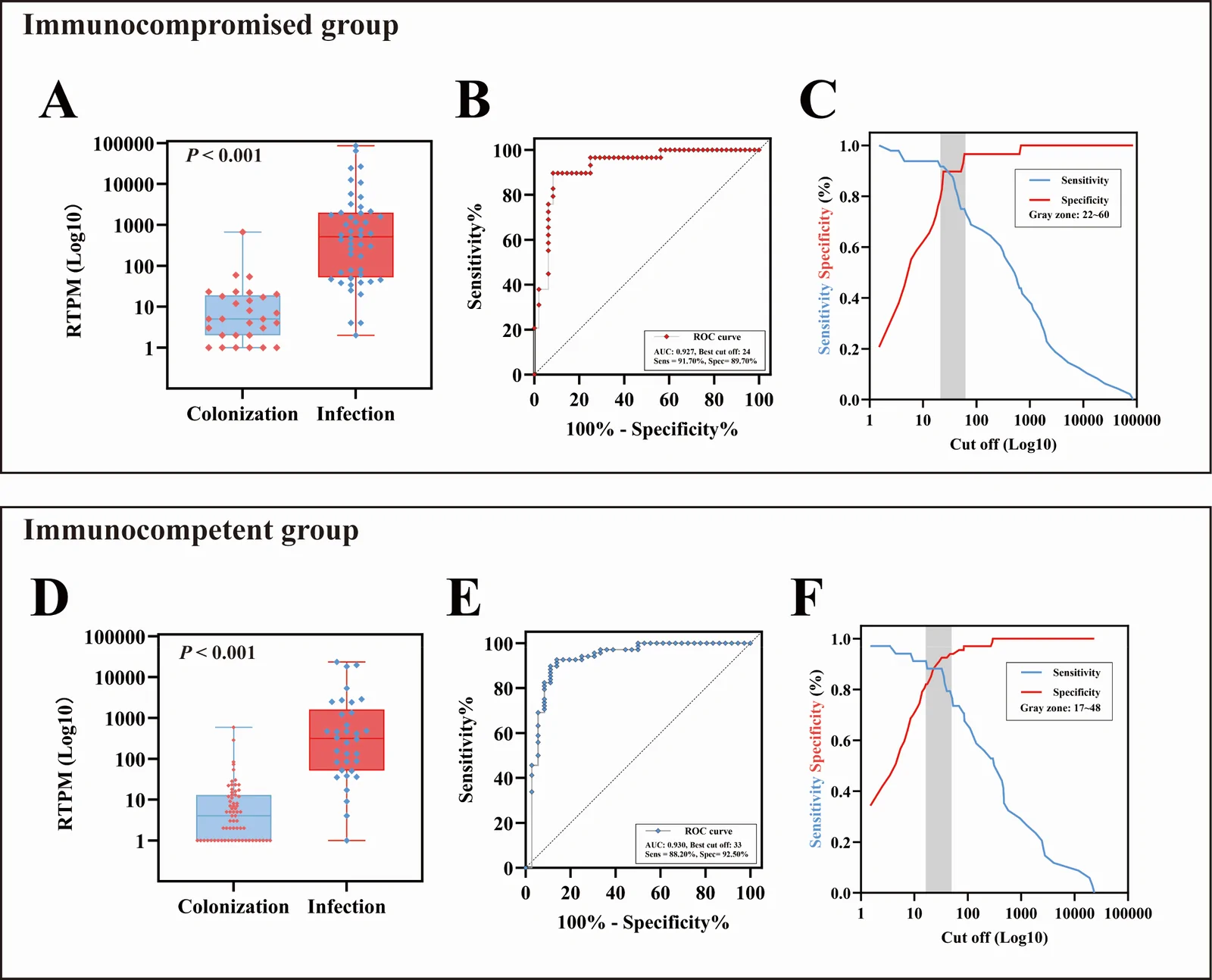
(**A**) Box plot of the immunocompromised group, (**B**) ROC curve analysis of RPTM values for distinguishing *Aspergillus* infection from colonization in the immunocompromised group, and (**C**) gray zone analysis to define the diagnostic uncertainty range. (**D**) Box plot of the immunocompetent group, (**E**) ROC curve analysis of RPTM values for distinguishing *Aspergillus* infection from colonization in the immunocompetent group, and (**F**) gray zone analysis to define the diagnostic uncertainty range. Note: In C and F, the shaded gray area indicates the diagnostic gray zone defined as the range of RPTM values for which either sensitivity or specificity does not reach 90%. Values within this interval are considered diagnostically indeterminate and should be interpreted together with clinical, radiological, and conventional microbiological findings.

In the immunocompromised group, RPTM values were significantly higher in infection cases compared with colonization (Fig. 2A). ROC curve analysis demonstrated that RPTM had good discriminatory ability for distinguishing *Aspergillus* infection from colonization among patients with Aspergillus-positive BALF-mNGS results, with an AUC of 0.927 (95% confidence interval [CI]: [0.867–0.988], *P* < 0.001) at an optimal cutoff value of 24, yielding a sensitivity of 91.70% and a specificity of 89.70% (Fig. 2B). Gray zone analysis further identified an indeterminate range (22 to 60), with 48.05% (37/77) of patients being in the gray zone (Fig. 2C).

In the immunocompetent group, RPTM values were also significantly higher in infection cases compared with colonization (Fig. 2E). ROC curve analysis demonstrated that RPTM had a good discriminatory ability for predicting IPA, with an AUC of 0.929 (95% CI: [0.870–0.987], *P* < 0.001) at an optimal cutoff value of 33, yielding a sensitivity of 88.20% and a specificity of 92.50% (Fig. 2D). Gray zone analysis further identified an indeterminate range (17 to 48), with 30.69% (31/101) of patients being in the gray zone (Fig. 2F).

### Characteristics and microbiota features related to *Aspergillus* infection in immunocompromised patients

**Fig 3:**
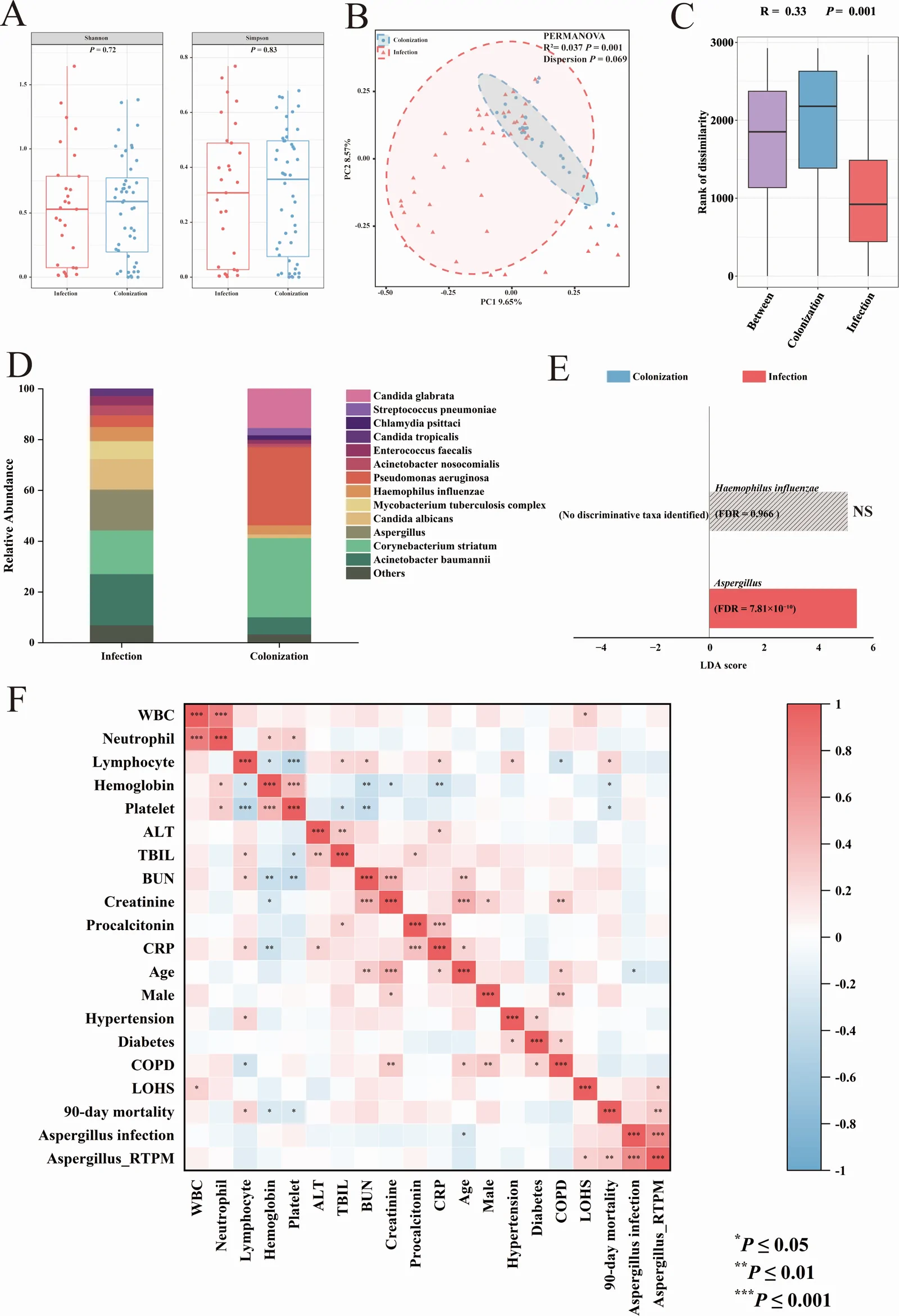
Microbiome analysis in immunocompromised group. (**A**) α diversity. (**B**) β diversity by PCoA. (**C**) β diversity by ANOSIM. (**D**) Top 10 species by relative abundance. (**E**) Differentially abundant microbes identified by LEfSe analysis (taxa showing significance in the initial LEfSe analysis but failing FDR correction are labeled as NS). (**F**) Spearman correlations between Aspergillus_RPTM and clinical characteristics.

In the immunocompromised group, α diversity analysis showed no significant differences between the infection and colonization groups (Shannon, *P* = 0.72; Simpson, *P* = 0.83) (Fig. 3A). β diversity analysis by PCoA showed limited visual separation between the two groups (PC1 = 9.65%, PC2 = 8.57%). Permutational Multivariate Analysis of Variance (PERMANOVA) confirmed significant differences in microbial community composition between infection and colonization groups (*R*² = 0.037, *P* = 0.001), while beta-dispersion analysis showed no significant difference in within-group dispersion (*P* = 0.069) (Fig. 3B).

The relative abundance of dominant taxa was visualized in bar plots (Fig. 3D). In the *Aspergillus* infection group, the top three most abundant microorganisms were *Acinetobacter baumannii*, *Corynebacterium striatum*, and *Aspergillus*, whereas in the *Aspergillus* colonization patients, they were *Corynebacterium striatum*, *Pseudomonas aeruginosa*, and *Candida albicans*. LEfSe analysis identified *Aspergillus* as a significantly enriched taxon in infection group (LDA score = 5.40, *P* = 5.92 × 10^−5^, FDR = 7.81 × 10^−10^). *Haemophilus influenzae* was identified as a discriminative taxon by LEfSe (LDA score = 5.07, *P* = 0.047), but did not remain significant after FDR correction (FDR = 0.966). No taxa met the criteria for significant enrichment in the colonization group (Fig. 3E).

Spearman correlation analyses showed that Aspergillus_RPTM was strongly associated with *Aspergillus* infection status (*R* = 0.72, *P* ≤ 0.001) and negatively correlated with age (*R* = −0.22, *P* ≤ 0.05). Additionally, Aspergillus_RPTM was positively correlated with 90-day mortality (*R* = 0.32, *P* ≤ 0.01) and LOHS (*R* = 0.28, *P* ≤ 0.05), suggesting a potential link between *Aspergillus* abundance and adverse clinical outcomes (Fig. 3F).

### Characteristics and microbiota features related to *Aspergillus* infection in immunocompetent patients

**Fig 4:**
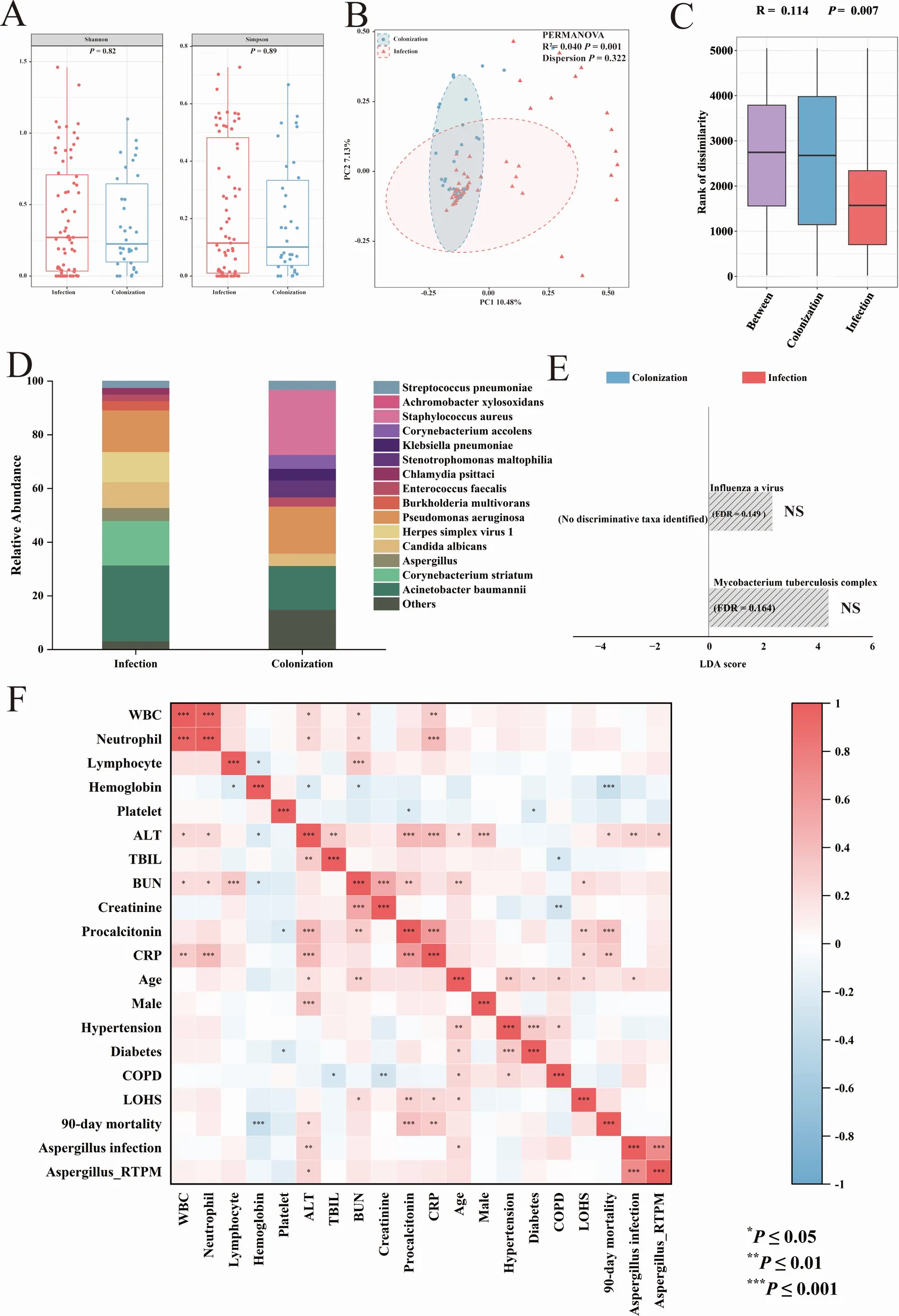
Microbiome analysis in immunocompetent group. (**A**) α diversity. (**B**) β diversity by PCoA. (**C**) β diversity by ANOSIM. (**D**) Top 10 species by relative abundance. (**E**) Differentially abundant microbes identified by LEfSe analysis (Taxa showing significance in the initial LEfSe analysis but failing FDR correction are labeled as NS). (**F**) Spearman correlations between Aspergillus_RPTM and clinical characteristics.

In the immunocompetent group, α diversity analysis showed no significant differences between the infection and colonization groups (Shannon, *P* = 0.82; Simpson, *P* = 0.89) (Fig. 4A). β diversity analysis by PCoA showed limited visual separation between the two groups (PC1 = 10.48%, PC2 = 7.13%). PERMANOVA demonstrated significant differences in microbial community composition between infection and colonization groups (*R*² = 0.040, *P* = 0.001), while beta-dispersion analysis showed no significant difference in within-group dispersion (*P* = 0.322) (Fig. 4B and C).

The relative abundance of dominant taxa was visualized in bar plots (Fig. 4D). In the infection patients, the top three most abundant microorganisms were *Acinetobacter baumannii*, *Corynebacterium striatum*, and *Pseudomonas aeruginosa*, whereas in the colonization patients, they were *Staphylococcus aureus*, *Pseudomonas aeruginosa*, and *Acinetobacter baumannii*. LEfSe analysis identified Influenza A virus (LDA score = 4.38, *P* = 0.014, FDR = 0.149) and the *Mycobacterium tuberculosis complex* (LDA score = 2.32, *P* = 0.046, FDR = 0.164) as discriminative taxa associated with infection group, although these taxa did not remain statistically significant after FDR correction. No taxa met the criteria for significant enrichment in the colonization group (Fig. 4E).

Further Spearman correlation analyses revealed that *Aspergillus* infection was strongly associated with Aspergillus_RPTM (R = 0.70, *P* ≤ 0.001) and positively correlated with ALT (R = 0.25, *P* ≤ 0.05) and age (R = 0.21, *P* ≤ 0.05). Apart from Aspergillus infection status, Aspergillus_RPTM was only significantly associated with ALT (R = 0.25, *P* ≤ 0.05), suggesting limited correlations with other clinical parameters (Fig. 4F).

### Clinical impact for BALF-mNGS

**Fig 5:**
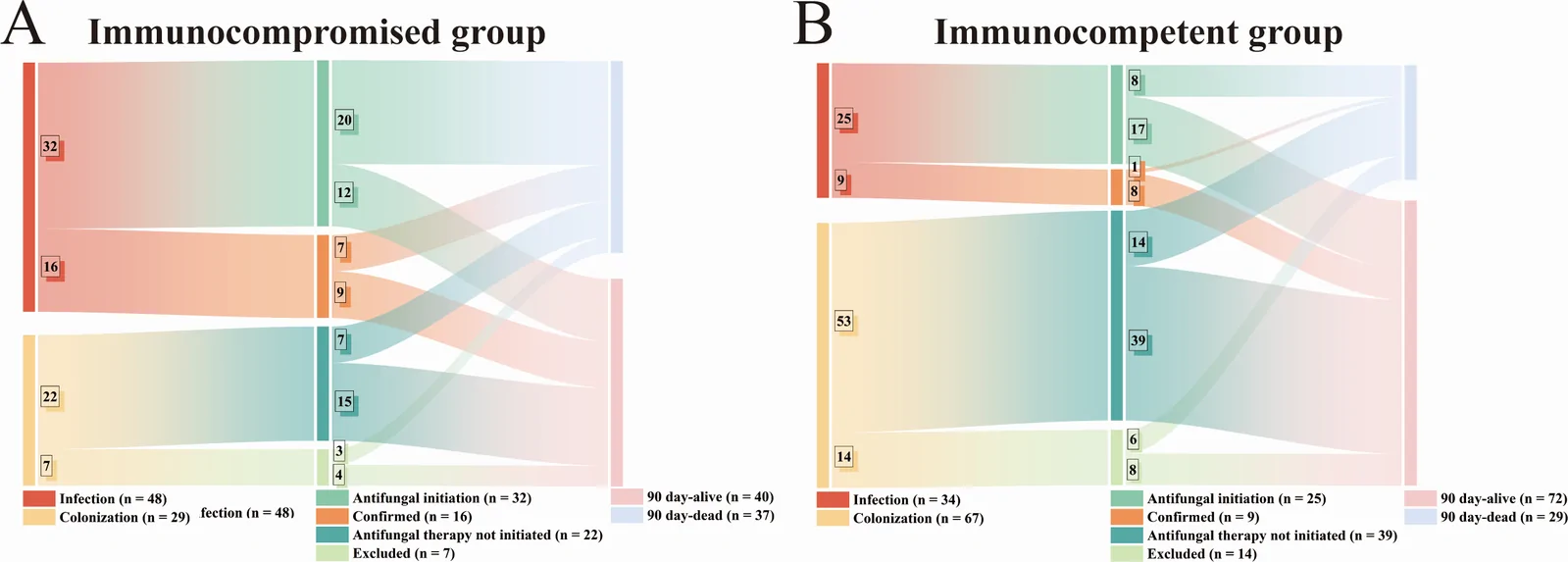
Potential clinical impact of BALF-mNGS on antifungal regimen adjustments and subsequent prognosis. Note: the Sankey diagram illustrates the relationship between *Aspergillus* infection status, antifungal treatment modification, and 90-day mortality. (**A**) The immunocompromised group (**B**) The immunocompetent group.

We used a Sankey diagram to visualize the potential clinical impact of BALF-mNGS on antifungal regimen adjustments in both groups. In the immunocompromised group, among patients with *Aspergillus* infection (*n* = 48), antifungal therapy was newly initiated (antifungal initiation) in 32 cases, with a mortality of 62.50% (20/32). Sixteen patients continued empirical antifungal treatment (confirmed), of whom 43.75% (7/16) died. The overall 90-day mortality in patients with *Aspergillus* infection was 56.25% (27/48). In colonization patients (*n* = 29), antifungal therapy was not initiated in 22 patients, with a mortality of 31.82% (7/22), and seven patients discontinued antifungal therapy (excluded), with a mortality of 42.86% (3/7). The overall 90-day mortality in colonization patients was 34.48% (10/29) (Fig. 5A).

In the immunocompetent group, among patients with *Aspergillus* infection (*n* = 34), antifungal therapy was newly initiated in 25 cases, with an overall 90-day mortality of 32.00% (8/25). Nine patients continued empirical antifungal treatment, of whom 11.11% (1/9) died. In colonization patients (*n* = 67), antifungal therapy was not initiated in 53 patients, with a mortality of 26.42% (14/53), and 14 patients discontinued antifungal therapy, with a mortality of 42.86% (6/14) (Fig. 5B).

**Fig 6:**
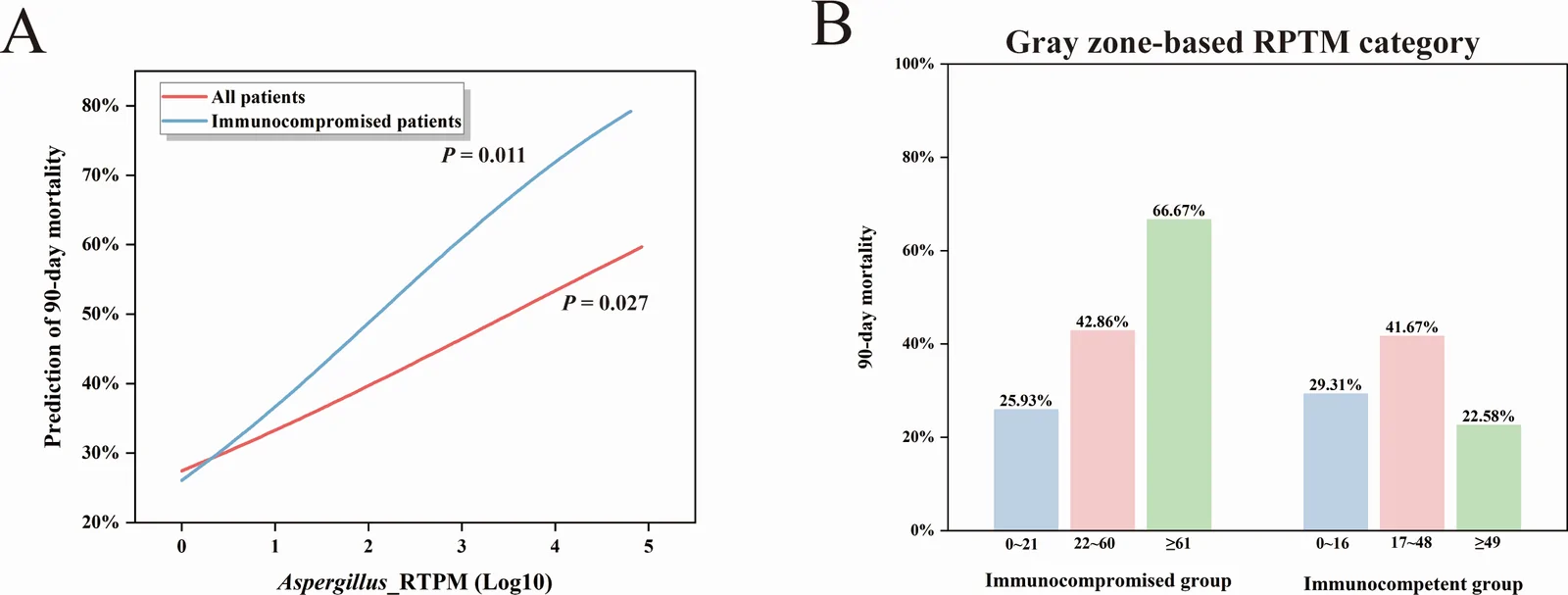
(**A**) Logistic regression analysis. (**B**) Association of Aspergillus_RPTM with 90-day mortality in both groups; for the immunocompromised group, the categories were RPTM ≤ 21, 22–60, and ≥61; for the immunocompetent group, they were RPTM ≤ 16, 17–48, and ≥49, corresponding to the gray-zone analysis shown in Fig. 2C and F.

We further performed logistic regression analysis to evaluate the association between Aspergillus_RPTM and 90-day mortality across different patient subgroups (Table 2). In all patients (*n* = 178), Aspergillus_RPTM was significantly associated with mortality (1.319 [1.033–1.684], *P* = 0.027). In the immunocompromised group (*n* = 77), the association was more pronounced (1.641 [1.118–2.410], *P* = 0.011). However, no significant association was observed in the immunocompetent group (0.916 [0.624–1.343], *P* = 0.653). When stratified by infection status, the RPTM was not significantly associated with mortality in either *Aspergillus* infection subgroup (1.248 [0.816–1.908], *P* = 0.308) or Aspergillus colonization subgroup (1.413 [0.727–2.747], *P* = 0.308). To better illustrate these results, we visualized the significant associations in all patients and the immunocompromised group. (Fig. 6A).

**TABLE 2:** Logistic regression analysis of the association between Aspergillus_RPTM and 90-day mortality across patient subgroups^*a*^

Patient subgroup	OR (95% CI)	*P*-value
All patients (*n* = 178)	1.319 (1.033–1.684)	0.027
Immunocompromised (*n* = 77)	1.641 (1.118–2.410)	0.011
Immunocompetent group (*n* = 101)	0.916 (0.624–1.343)	0.653
*Aspergillus* infection (*n* = 82)	1.248 (0.816–1.908)	0.308
*Aspergillus* colonization (*n* = 96)	1.413 (0.727–2.747)	0.308

^*a*^ Note: ORs were estimated using univariable logistic regression, with log10-transformed Aspergillus_RPTM as a continuous variable.

In addition, patients were stratified according to the RPTM gray-zone range to examine its relationship with 90-day mortality (Fig. 2C and E). The results showed that in the immunocompromised group, mortality increased with higher RPTM values, whereas no such trend was observed in the immunocompetent group (Fig. 6B).

## DISCUSSION

In this study, we investigated the application of BALF-mNGS in patients with suspected IPA under different immune statuses, focusing on three aspects: distinguishing infection from colonization, characterizing the pulmonary microbiome, and evaluating clinical impact. Our findings indicate that quantitative BALF-mNGS provides additional diagnostic information for distinguishing *Aspergillus* infection from colonization, reveals differences in pulmonary microbial composition, and may offer potential prognostic information, particularly in immunocompromised patients.

IPA remains a major cause of morbidity and mortality, especially in immunocompromised patients, but it also occurs in selected immunocompetent individuals with severe respiratory illness (23, 24). Differentiating true infection from colonization is a persistent challenge, as conventional microbiological tests lack sufficient sensitivity and specificity. In this context, mNGS offers an improved diagnostic approach by providing comprehensive pathogen detection and microbial community profiling (13, 25–27). These studies have demonstrated the value of mNGS in detecting *Aspergillus* infections; however, few have attempted to quantitatively interpret mNGS results to differentiate colonization from true infection, as the interpretation of mNGS findings remains challenging and often requires integration with conventional methods and clinical judgment. To date, only one published study by Ziwei Jiang et al. (16) has attempted to quantitatively interpret BALF-mNGS results to distinguish *Aspergillus* infection from colonization, identifying an optimal cut-off of 23 reads by ROC analysis (sensitivity: 80.56%, specificity: 90.63%). However, their study did not stratify patients according to immune status, even though immune status is a well-recognized risk factor and critical determinant in the development and progression of IPA (25, 28).

Our study further refined these findings by stratifying patients according to immune status and demonstrated comparable results. Specifically, in both immunocompromised and immunocompetent groups, Aspergillus_RPTM values were significantly higher in patients with infection than in those with colonization (Fig. 2A and D). ROC curve analysis identified an optimal cut-off value of 24 in the immunocompromised group (AUC: 0.927, sensitivity: 91.7%, specificity: 89.7%) (Fig. 2B) and 33 in the immunocompetent group (AUC: 0.930, sensitivity: 88.2%, specificity: 92.5%) (Fig. 2E). The different optimal thresholds between immunocompromised and immunocompetent patients may reflect differences in host–pathogen interactions (1). Immunocompromised patients often have impaired innate and adaptive antifungal immunity, allowing *Aspergillus* to germinate and invade pulmonary tissues despite relatively limited fungal proliferation (29, 30). Therefore, a lower RPTM threshold may be sufficient to distinguish infection from colonization in this population. In contrast, immunocompetent individuals generally maintain stronger antifungal defenses, and higher *Aspergillus* burdens may be required before clinically significant infection develops.

While ROC analysis determines optimal cut-off values, it does not account for uncertainty near the threshold. Therefore, we further performed a gray-zone analysis to better reflect clinical practice. Quantitative BALF-mNGS enabled improved discrimination between infection and colonization. ROC analysis defined optimal cut-off values of 24 and 33 reads for immunocompromised and immunocompetent patients (Fig. 2B and E), respectively, while gray-zone analysis delineated ranges of diagnostic uncertainty (22–60 and 17–48), guiding clinical interpretation in cases near the threshold (Fig. 2C and F). This approach allows clinicians to confidently distinguish infection from colonization outside the gray zone, while cases within the gray zone require integration of clinical judgment, conventional tests, and radiological findings. Notably, stratifying patients by immune status provides more precise guidance than previous studies that did not account for immune differences.

Beyond its diagnostic role, BALF-mNGS provides additional insights into the pulmonary microbiome, which may further inform clinical management and prognostic assessment. Previous studies have reported decreased α diversity and enrichment of specific bacterial taxa, including *Staphylococcus*, *Escherichia*, *Paraclostridium*, and *Finegoldia*, alongside reductions in *Prevotella* and *Veillonella* (31). Consistent with these observations, we found distinct microbial community structures in immunocompromised patients, with *Aspergillus* identified by LEfSe as the only taxon remaining significant after FDR correction, whereas α diversity remained unchanged (Fig. 3A and E). In immunocompetent patients, PERMANOVA demonstrated differences in microbial community composition between infection and colonization groups (*R*² =0.040, *P* = 0.001) despite limited visual separation in PCoA. LEfSe identified Influenza A virus and the *Mycobacterium tuberculosis* complex as candidate taxa, but neither remained significant after FDR correction (Fig. 4B and E); however, no significant correlation was detected between Aspergillus_RPTM and clinical outcomes. These findings suggest that *Aspergillus* infection may primarily involve selective expansion of specific pathogenic taxa rather than a global disruption of pulmonary microbial diversity. The absence of significant α-diversity changes indicates that IPA-related microbial alterations may be driven by changes in community composition rather than overall species richness or evenness. Furthermore, the stronger association between *Aspergillus* abundance and clinical outcomes observed in immunocompromised patients suggests that quantitative *Aspergillus* burden may have greater prognostic relevance in hosts with impaired antifungal immunity.

Taken together, these findings suggest that *Aspergillus* infection primarily alters microbial composition rather than overall diversity. This contrasts with previous reports of decreased α diversity in IPA patients (31), likely reflecting differences in patient populations, inclusion criteria, or sequencing methodologies.

Previous studies have highlighted the clinical utility of BALF-mNGS in IPA. A multicenter retrospective study demonstrated that mNGS-guided antimicrobial adjustments significantly reduced 28-day mortality in immunocompromised critically ill patients with IPA (13). Another study on community-acquired pneumonia patients with IPA showed higher hospital and ICU mortality, with lung microbiome features correlating with clinical outcomes (32). In addition, a study in diabetic patients showed that 47.6% of IPA cases had their initial antimicrobial therapy modified based on mNGS results, demonstrating the potential of mNGS to guide treatment decisions (33). In our study, mNGS provided additional microbiological information that may assist antifungal decision-making (Fig. 5A and B). In both groups, mNGS provided additional microbiological evidence that supported antifungal initiation or continuation in patients with *Aspergillus* infection. It also provided information that may help avoid unnecessary antifungal exposure in patients classified as colonization. The higher mortality observed in immunocompromised patients likely reflects their greater underlying vulnerability and disease complexity. These findings suggest that BALF-mNGS may assist antifungal decision-making by providing additional microbiological information, although its impact on clinical outcomes requires prospective validation.

Compared with established fungal biomarkers, such as BALF GM and PCR, quantitative BALF-mNGS provides a broader assessment of microbial nucleic acid abundance and enables simultaneous detection of multiple pathogens. BALF GM and PCR remain important tools for IPA diagnosis, but their interpretation may be affected by factors, such as antifungal exposure, colonization, and differences in assay standardization. In contrast, RPTM provides a quantitative measure of *Aspergillus* sequence abundance, which may serve as a surrogate marker of fungal burden. However, RPTM does not directly reflect fungal viability or tissue invasion and should, therefore, be interpreted together with clinical, radiological, and conventional microbiological findings.

To further explore prognostic implications, logistic regression revealed that higher Aspergillus_RPTM was significantly associated with increased 90-day mortality in immunocompromised patients, but not in immunocompetent patients (Table 2). Stratification according to the RPTM gray zone corroborated this trend, with mortality increasing alongside higher RPTM values in immunocompromised hosts (Fig. 6A and B). The stronger association between Aspergillus_RPTM and mortality in immunocompromised patients may be explained by the closer relationship between fungal burden and host vulnerability in this population. In immunocompromised patients, increased *Aspergillus* burden may reflect uncontrolled fungal proliferation due to impaired host defense, thereby indicating more severe infection and a higher risk of adverse outcomes (34). Conversely, mortality in immunocompetent patients may be influenced by multiple competing factors, including underlying pulmonary disease, bacterial co-infections, and critical illness severity, which may attenuate the association between *Aspergillus* burden and clinical outcomes. These findings suggest that quantitative BALF-mNGS may be associated with prognostic differences and could potentially contribute to risk stratification, particularly in immunocompromised patients, although this potential role requires validation in larger prospective cohorts.

## Limitation

First, although this was a multicenter study, the sample size remained relatively limited due to the rarity of the target population. This restricted the construction of fully adjusted multivariable models and external validation of the proposed RPTM cutoff and gray-zone thresholds. Therefore, the association between Aspergillus_RPTM and 90-day mortality should be interpreted as an association rather than evidence of an independent predictive effect. Second, the retrospective design may have introduced unmeasured confounding and selection bias. In addition, although BALF-mNGS provides quantitative information on *Aspergillus* abundance, RPTM reflects sequence abundance rather than viable fungal burden or tissue invasion and should, therefore, be interpreted together with clinical, radiological, and conventional microbiological findings. Third, we did not perform microbial interaction analyses between *Aspergillus* and other pulmonary microorganisms, as BALF-mNGS data are compositional and sparse, and reliable microbial network inference requires dedicated analytical approaches and larger validation cohorts. Finally, inter-laboratory variability in RPTM standardization and the cost, turnaround time, and interpretation complexity of BALF-mNGS may limit its broader clinical application.

## Conclusion

Quantitative BALF-mNGS RPTM may assist in differentiating *Aspergillus* infection from colonization among patients with Aspergillus-positive BALF-mNGS results and reveals microbial community shifts, particularly in immunocompromised patients. It guides targeted antifungal therapy, avoiding unnecessary treatment, and higher Aspergillus_RPTM values were associated with increased 90-day mortality in immunocompromised hosts in this exploratory analysis. These findings support the potential role of quantitative BALF-mNGS in diagnostic assessment and risk stratification of IPA.

## Data Availability

The raw sequence data for this study have been uploaded and deposited in NCBI (BioProject No. PRJNA1333653). More deidentified data are available from the corresponding author upon reasonable request.

## SUPPLEMENTAL MATERIAL

Table S1 spectrum.01045-26-s0001.docx Clinical, radiological, and microbiological evidence supporting the final diagnosis of IPA.

ASM does not own the copyrights to Supplemental Material that may be linked to, or accessed through, an article. The authors have granted ASM a non-exclusive, world-wide license to publish the Supplemental Material files. Please contact the corresponding author directly for reuse.
