# Supplementary material for: Clinical application of BALF-mNGS in immunocompromised and immunocompetent patients with suspected invasive pulmonary aspergillosis: differentiating infection from colonization, microbiome features, and clinical impact
Source: Microbiol Spectr. 2026 Aug 28;14(10):e01045-26. doi: 10.1128/spectrum.01045-26 (PMC13689266; doi:10.1128/spectrum.01045-26)
Supplement: Supplementary file 1 [file spectrum.01045-26-s0001.docx]

**Supplementary Table S1.** Clinical, radiological, and microbiological evidence supporting the final diagnosis of IPA.

|  | Immunocompromised Infection (n= 48) | Immunocompromised Colonization (n= 29) | Immunocompetent Infection (n= 34) | Immunocompetent Colonization (n= 67) |
| --- | --- | --- | --- | --- |
| Sputum culture positive for *Aspergillus* | 12 (25.0%) | 8 (27.6%) | 9 (26.5%) | 11 (16.4%) |
| Serum GM positive | 18 (37.5%) | 3 (10.3%) | 10 (29.4%) | 5 (7.5%) |
| (1,3)-β-D-glucan test | 13 (27.1%) | 2 (6.9%) | 7 (20.6%) | 3 (4.5%) |
| BALF culture positive for *Aspergillus* | 14 (29.2%) | / | 12 (35.3%) | / |
| PCR | 9 (18.8%) | / | 6 (17.7%) | / |
| Histopathology positive | 3 (6.3%) | / | 4 (11.8%) | / |
| Diagnosis based on clinical and radiological assessment | 7 (14.6%) | / | 11 (32.6%) | / |

The final diagnosis was established according to the EORTC/MSSGERC 2020 criteria in immunocompromised patients and multidisciplinary expert adjudication integrating clinical, radiological, and microbiological findings in immunocompetent patients.

**Note:** Due to the retrospective design, diagnostic tests other than BALF-mNGS were not uniformly performed in all patients. Thus, positivity rates represent the proportion of positive results among tested patients rather than the true positivity rates of these assays.
